# Next-Generation Sequencing Analysis Reveals Novel Pathogenic Variants in Four Chinese Siblings With Late-Infantile Neuronal Ceroid Lipofuscinosis

**DOI:** 10.3389/fgene.2019.00370

**Published:** 2019-04-25

**Authors:** Xiao-Tun Ren, Xiao-Hui Wang, Chang-Hong Ding, Xiang Shen, Hao Zhang, Wei-Hua Zhang, Jiu-Wei Li, Chang-Hong Ren, Fang Fang

**Affiliations:** ^1^Department of Neurology, National Centre for Children's Health, Beijing Children's Hospital, Capital Medical University, Beijing, China; ^2^Running Gene Inc., Beijing, China

**Keywords:** Next-Generation Sequencing (NGS), Neuronal Ceroid Lipofuscinosis (NCL), late-infantile, CLN2, CLN5, CLN6, CLN7

## Abstract

Neuronal Ceroid Lipofuscinoses (NCLs) are progressive degenerative diseases mainly affect brain and retina. They are characterized by accumulation of autofluorescent storage material, mitochondrial ATPase subunit C, or sphingolipid activator proteins A and D in lysosomes of most cells. Heterogenous storage material in NCLs is not completely disease-specific. Most of CLN proteins and their natural substrates are not well-characterized. Studies have suggested variants of Late-Infantile NCLs (LINCLs) include the major type CLN2 and minor types CLN5, CLN6, CLN7, and CLN8. Therefore, combination of clinical and molecular analysis has become a more effective diagnosis method. We studied 4 late-infantile NCL siblings characterized by seizures, ataxia as early symptoms, followed by progressive regression in intelligence and behavior, but mutations are located in different genes. Symptoms and progression of 4 types of LINCLs are compared. Pathology of LINCLs is also discussed. We performed Nest-Generation Sequencing on these phenotypically similar families. Three novel variants c.1551+1insTGAT in TPP1, c.244G>T in CLN6, c.554-5A>G in MFSD8 were identified. Potential outcome of the mutations in structure and function of proteins are studied. In addition, we observed some common and unique clinical features of Chinese LINCL patient as compared with those of Western patients, which greatly improved our understanding of the LINCLs.

## Introduction

NCLs (Neuronal Ceroid Lipofuscinoses) are characterized by accumulation of lysosomal autofluorescent storage material and progressive neurodegeneration (Oishi et al., 1999; Getty and Pearce, 2011; Blom et al., 2013; Kollmann et al., 2013; Mink et al., 2013; Sands, 2013; Patino et al., 2014). The most common clinical features of NCLs include epileptic seizures, progressive regression of intelligence, loss of motor function, retinal degeneration, and premature death (Haltia and Goebel, 2013; Mink et al., 2013; Warrier et al., 2013; De Silva et al., 2015). NCL is one of the most frequent classes of childhood-onset neurodegenerative diseases with prevalence around 0.5–8 per 100,000 live births varying by the regions (Oishi et al., 1999; Getty and Pearce, 2011; Cotman et al., 2013; Haltia and Goebel, 2013; Beltran et al., 2018). So far there are 13 genes identified as candidate genes of NCLs, i.e., *CLN1/PPT1, CLN2/TPP1, CLN3, CLN4/DNAJC5, CLN5, CLN6, CLN7/MFSD8, CLN8, CLN10/CTSD, CLN11/GRN, CLN12/ATP13A2, CLN13/CTSF*, and *CLN14/KCTD7* (Kollmann et al., 2013; Warrier et al., 2013). Most human NCLs follow autosomal recessive model except that caused by *CLN4/DNAJC5* gene which presents autosomal dominant form (Haltia and Goebel, 2013; Kollmann et al., 2013; Warrier et al., 2013). However, the storage material of NCLs is not disease-specific and the function of most CLN protein has not been well classified. It has been difficult to diagnose merely based on clinical findings. Enzyme assay is used to help diagnose NCLs with mutations in enzyme-coding genes, *CLN1/PPT1, CLN2/TPP1, CLN10/CTSD* with deficiency of respective enzymes (Kamate et al., 2012; Mole and Williams, 2013). Another enzyme-coding genes, *CLN13/CTSF*, as a novel candidate gene identified in year 2013 is also presumably to cause impairment in enzyme cathepsin F (Mole and Williams, 2013; Schulz et al., 2013), but no supportive experiment due to few cases. Merely based on clinical presentations, NCL could not be easily distinguished from other diseases including Leber's Hereditary Optic Neuropathy (LHON), the symptoms of which include sezures, regression, ataxia, and vision impairment (Fang et al., 2017). Due to limitations of other methods, sequencing has emerged as an effective diagnosis method.

By onset ages, NCLs are classified into congenital, infantile, late-infantile, juvenile, and adult NCLs. Late-Infantile NCLs (LINCLs) include the classic CLN2 disease and variant CLN5, CLN6, CLN7, and CLN8 disease (Getty and Pearce, 2011; Mole and Williams, 2013; Schulz et al., 2013; Warrier et al., 2013; Patino et al., 2014). In this study, we present four families with CLN2, CLN5, CLN6, and CLN7 disease, respectively.

CLN2 disease caused by mutations in *CLN2* gene which encodes the tripeptidyl peptidase 1, a lysosomal serine protease that removes tripeptides from N-terminus of peptides (Kollmann et al., 2013). Mitochondrial ATP-synthase subunit C, a significant component of storage material in LINCL has been demonstrated as one of the substrates of TPP1 (Ezaki et al., 2000). Deficiency of TPP1 activity results in accumulation of mitochondrial ATP-synthase subunit C, which may be the pathology of LINCL.

*CLN5* gene encodes a soluble polypeptide which predominantly colocalizes with lysosomal-associated membrane protein-1 (LAMP1). Mutations in *CLN5* may cause retention in the ER/Golgi (Isosomppi et al., 2002; Lebrun et al., 2009; Schmiedt et al., 2010). Since CLN5 is a highly glycosylated protein, it may play an essential role as a sensor in trafficking or integrity of lysosomes (Kollmann et al., 2013).

*CLN6* gene encodes a highly conserved membrane protein that exclusively resides in endoplasmic reticulum (ER) (Heine et al., 2004; Kollmann et al., 2013). However, the exact function of CLN6 is still unknown. Previous experiment shows that loss of CLN6 activity may affect lysosomal degradation of Arylsulfatase A (Heine et al., 2004). This finding may indicate CLN6 could play a role in degradation involving ER. Besides, CLN6 was also proved to interact with Collapsin Response Mediator Protein-2 (CRMP-2). This interaction probably affects maturation and integrity of axonal outgrowth thus contribute to neuronal dysfunction of LINCL patients (Benedict et al., 2009).

*CLN7/MFSD8* encodes a lysosomal membrane protein called Major Facilitator Superfamily Domain-containing protein 8 (MFSD8). This protein is ubiquitously expressed with several splicing variants. It could transport small solutes by electrochemical gradients (Siintola et al., 2007). However, the specific substrates of CLN7 require further investigation.

In the present study, we found three novel mutations of LINCLs. They are likely pathogenic by analyzing their functional consequences and correlation with the phenotypes. In addition, we also observed some common and unique clinical features of Chinese LINCL patient as compared with those of Western patients, which may improve understanding of the LINCLs.

## Case Presentation

**Figure 1:**
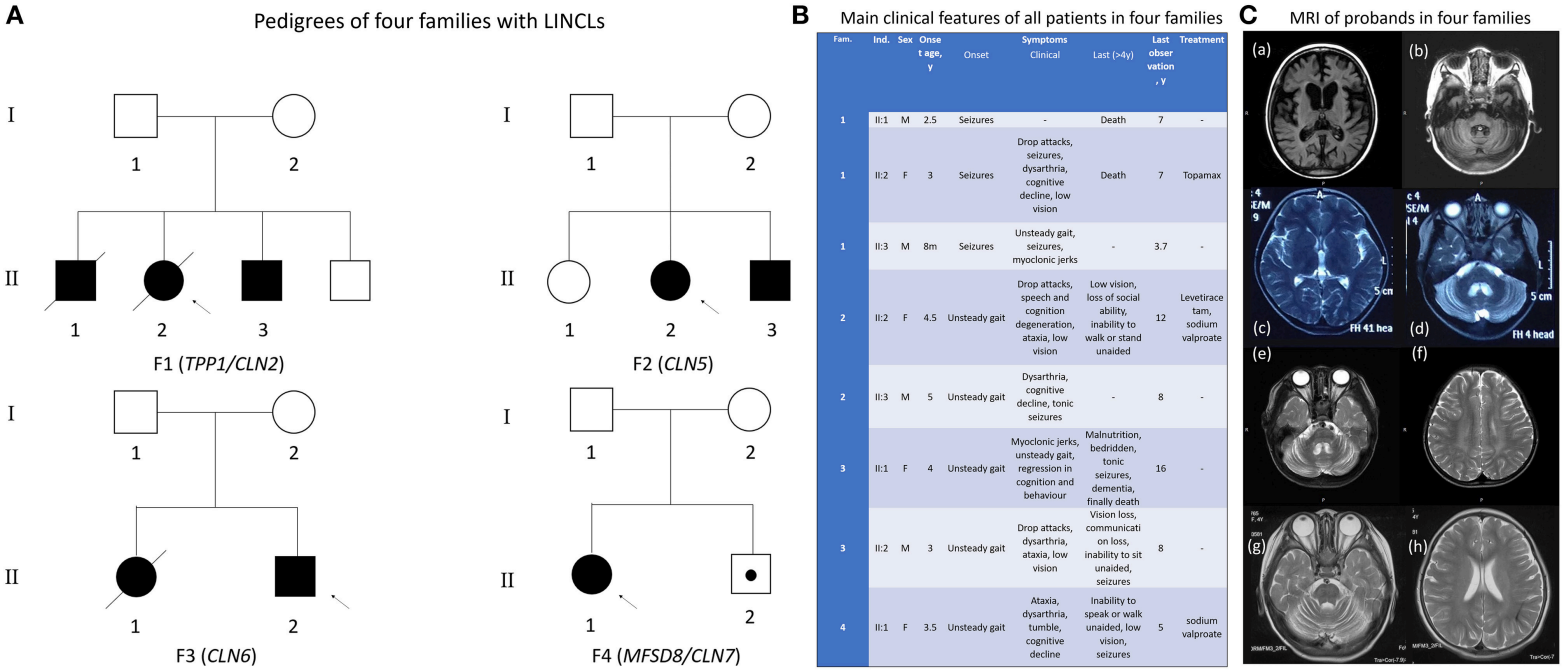
Clinical information of all patients in four families. **(A)** Pedigrees of the four families. Proband (F1 II:2) and proband's elder brother (F1 II:1) in family 1 and proband's elder sister (F3 II:1) in family 3 passed away. The little brother of proband in family 4 (F4 II:2) was just 1 year old and did not reach the general onset age, 3–4.5 years old. However, he was identified with the same pathogenic gene as his sister, the proband of family 4. **(B)** Overview of the main clinical features of all patients. Clinical course of all patients is nearly identical except for the earlier onset age and different initial symptom in family 1. **(C)** MRI images from 4 probands demonstrating severe cerebellar atrophy in all patients. Atrophy in cerebrum and brain stem was observed in proband 1 of family 1 (a, b). Abnormal myelination in white matter was found in proband 3 of family 3.

Eight patients including four probands were born in four healthy non-consanguineous Chinese families with normal pregnancy and perinatal history. Pedigrees of four families were presented in Figure 1A. All four families possess unremarkable family history. Typically, unsteady gait was observed between age 3–5 as the initial symptom. Only in Family one, seizures were observed as the first symptom at slightly earlier ages from 8 months to 3 years old. Regression in cognition and behavior were then observed in all affected children. Ataxia, seizures were also presented in all patients. Low vision was found in a few patients at this stage. At later stage, most patients lost the ability to sit, stand or walk unaided, they also lost their vision. Two patients in family one died at age seven, one patient in family three died at age 16 (for a comparison of all patients see Figure 1B).

Cerebellar atrophy was confirmed by MRI imaging in all four probands. Proband in family 1 also had very significant cerebral atrophy accompanied by atrophy of brain stem, while proband in family three had abnormal myelination (Figure 1C). Only symptomatic treatment was used for patients, whereas no obvious improvement was observed. Proteolytic activity of TPP1 was completely lost in proband 1 (individual II:2 of family 1). The precise information of patients was collected in Table S1. All phenotypes of patients was standardized as HPO terms.

Proband 1 (II:2 of family 1) presented general epileptic discharges accompanied by burst-suppression. For proband 2 (II:1 of family 2), EEG background rhythm was slow, general medium to high amplitude slow waves, transient, or continuous slow spike-and-waves was observed. EEG of proband 3 (II:2 of family 3) showed frequent spike-and-wave and slow spike-and-wave discharged during the sleep period in right central electrode. For proband 4 (II:1 of family 4), massive epileptic discharges were noticed.

Since nearly all the probands have siblings who presents similar phenotypes, Whole-Exome Sequencing was performed to investigate the molecular genetic basis of the disease in these family. Sanger sequencing was performed to confirm the identified mutations.

Informed consents for genetic analyses were obtained from the children's parents. The study was approved by the ethics committee of Beijing Children's Hospital. Written informed consent was obtained from the patients' parents for the publication of this report and any accompanying images.

## Methods

### Reference Sequence

All positions in the mutated genes are annotated to reference sequence, namely RefSeq NM_000391, Ensembl ENST00000299427 for TPP/CLN2; NM_006493, Ensembl ENST00000377453 for CLN5; NM_017882, Ensembl ENST00000249806 for CLN6, NM_152778, Ensembl ENST00000296468 for MFSD/CLN7 in this publication. Whole Exome Sequence data were mapped and aligned to Human Genome Build GRCh37/hg19.

### Next-Generation Sequencing

Proband DNA was sequenced to discover the causal gene. DNA was isolated from peripheral blood using DNA Isolation Kit (Bioteke, AU1802). One microgram of genomic DNA was fragmented into 200–300 bp length by Covaris Acoustic System. The DNA fragments were then processed by end-repairing, A-tailing and adaptor ligation, a 4-cycle pre-capture PCR amplification, targeted sequences capture. Captured DNA fragments were eluted and amplified by 15 cycle post capture PCR. The final products were sequenced with 150-bp paired-end reads on Illumina HiSeq X platform according to the standard manual. The raw data produced on HiSeq X were filtered and aligned against the human reference genome (hg19) using the BWA Aligner (http://bio-bwa.sourceforge.net/). The quality recalibration was performed using GATK Base Recalibrator(Genome Analysis ToolKit) (www.broadinstitute.org/gatk). The single-nucleotide polymorphisms (SNPs) and small insertions or deletions (indel) were called by GATK Unified Genotyper (Genome Analysis ToolKit) (www.broadinstitute.org/gatk). Variants were annotated using ANNOVAR (annovar.openbioinformatics.org/en/latest/).

### Method of Mapping, Genotype, SNP Calling, and Indel Calling

Image analysis and base calling were performed using the Illumina Pipeline. BWA Aligner (http://bio-bwa.sourceforge.net/) was used to align clean reads to human reference genome (hg19), the parameters were set as default. The alignment result was then passed to GATK to identify the breakpoints, the parameters were set as “mismatch Fraction = 0.05, lod = 5, masReadsF or Realignment = 30,000, maxReadsInRam = 1,000,000.”

We selected variations obtained from exome sequencing with minor allele frequencies <0.05 in any of the following databases (dbSNP, Hapmap, 1000 Genomes Project). Effects of single-nucleotide variants (SNVs) were predicted by SIFT, Polyphen-2, and MutationTaster programs. All variants were interpreted according to ACMG standards and categorized to be pathogenic, likely pathogenic, variants of unknown clinical significance (VUS), likely benign, and benign. We further compared the rest of the deleterious variations in the patients with their unaffected parents and investigated the function of all identified genes according to the published reports and OMIM database.

### Sanger Sequencing

The candidate causal genes discovered via WES were then confirmed by Sanger sequencing and co-segregation analyses among the family were also conducted. The primers were designed using Primer Premier 5.0 (Premier Biosoft) and PCR was carried out to amplify the fragments covering the mutated sites. The PCR products were further purified with Zymoclean PCR purification Kit and then sequenced by ABI 3730 DNA Sequencer (Applied Biosystems, Foster City, CA, United States). Sanger sequencing results were analyzed by Chromas Lite v2.01 (Technelysium Pty Ltd., Tewantin, QLD, Australia).

## Result

### Variants Identified by Whole Exome Sequencing

Next-Generation Sequencing was carried out in exome of probands. All four probands were identified with mutations in *TPP1/CLN2, CLN5, CLN6*, and *MFSD8/CLN7*, respectively (Table 1, Figure S1). Among these seven mutations, c.1551+1insTGAT of *TPP1* gene, c.244G>T of *CLN6* and c.554-5A>G of *MFSD8* gene are novel mutations that haven't been reported before. According to ACMG guidelines, mutation c.1551+1insTGAT of *TPP1* was interpreted as pathogenic since this mutation is a null variant (PVS1), with extremely low frequency (PM2), and the phenotype of the patient is specific for the disease that related to the gene (PP4). Mutation c.244G>T of *CLN6* can be classified as likely pathogenic since it is absent from controls (PM2), was detected in trans with a recently reported pathogenic variant c.892G>A (PM3) co-segregated in multiple affected family members (PP1), predicted to be deleterious by multiple lines of computational evidence (PP3) i.e., predicted to be damaging by SIFT with score 0, deleterious by PROVEAN with score −5.92, probably damaging by PolyPhen-2 with score 1.00, disease causing by MutationTaster with score >0.99, the phenotype of the patient was also specific for disease CLN6 (PP4). Intronic variant c.554-5A>G of *MFSD8* was predicted to be VUS (variants of uncertain significance) according to ACMG standard, since this variant is absent from controls (PM2), detected in trans with a pathogenic variant c.1444C>T (PM3), and the phenotype of patient was similar to CLN7 (PP4).

**Table 1:** Gene sequencing result of 4 patients with NCL.

**Proband**	**Orientation**	**Inheritance**	**Gene**	**Pathogenic variants**
1	homozygous	AR	TPP1	c.1551+1insTGAT aberrant splicing of Exon 12
2	compound heterozygous	AR	CLN5	c.1068_1069del (Xin et al., 2010) p.(L358AfsX4) c.1100_1103del (Kohan et al., 2008) p.(K368SfsX15)
3	compound heterozygous	AR	CLN6	c.244G>T p.(G82W) c.892G>A (Sun et al., 2018) p.(E298K)
4	compound heterozygous	AR	MFSD8	c.1444C>T (Aiello et al., 2009) p.(R482X) c.554-5A>G aberrant splicing of Exon7

*Overview of the gene sequenced by next-generation sequencing in 4 probands*.

### Sanger Sequencing

All variants identified in Next-Generation Sequencing were then confirmed in other family members by Sanger Sequencing. Both parents of probands are the carriers of one of the two mutations, respectively. Other patients in the family also carry the same mutation as the proband. All results are shown in Figure S2.

## Discussion

NCLs are a group of neurological diseases without typical clinical symptoms. The symptoms of NCLs could not be well-distinguished from other neurological diseases especially at early stage. Traditional enzymatic activity detection can only determine certain types of NCLs, i.e., CLN1, CLN2, and CLN10. Other types of NCLs cannot be well-diagnosed until gene sequencing was introduced (Patino et al., 2014). Here we performed Whole-Exome Sequencing in four Chinese siblings with LINCLs, mutations in CLN genes were identified in all families including 3 novel mutations. Genetic test especially Whole-Exome Sequencing is now a suggestive tool in diagnosis of rare disease and is accepted and recommended by more clinicians now (Jin et al., 2018; Shen, 2018).

### Pathogenesis of Novel Mutations

In this study, 3 novel mutations in TPP1/CLN2, CLN6, and CLN7 were found, respectively. According to ACMG guidelines, mutation c.1551+1insTGAT in TPP1/CLN2 was characterized as pathogenic, mutation c.244G>T (G82W) in CLN6 was interpreted as likely pathogenic and mutation c.554-5A>G was classified as VUS.

**Figure 2:**
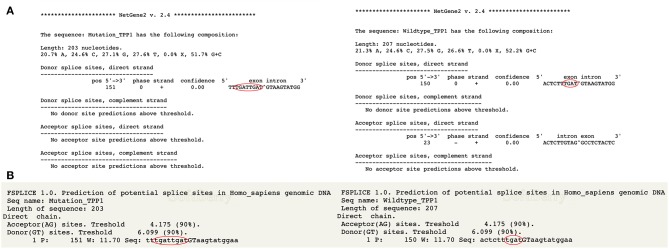
Prediction result of c.1551+1insTGAT by NetGene Server 2 (Brunak et al., 1991; Hebsgaard et al., 1996) and Softberry. **(A)** According to the result of NetGene Server 2, this variant could not alter the donor splice site but insert 4 nucleotides before the exon and intron border. **(B)** Result of Softberry (http://www.softberry.com) presents the same result that this variant is an insert variant rather than splicing variant.

For mutation c.1551+1insTGAT, *in silico* analysis suggests that this variant is a four-nucleotide-insertion in exonic region (Figure 2) It is probably not a splicing variant but an insertion variant which affects all the downstream sequence. Insertion of these 4 nucleotides would cause nonsense variant p.V518X. Resulted protein will be truncated after the residue D517. Residue from 518 to 563 would be missing. The highly conserved Ca-binding loop in sedolisin family (aa517-547) (Wlodawer et al., 2001, 2003) would be destroyed. Ca^2+^ is the cofactor of enzyme TPP1 and it was demonstrated necessary for the autocatalysis of the precursor TPP1 into mature form (Kuizon et al., 2010). Destruction of Ca-binding loop would disrupt the Ca^2+^ binding and sequentially hinder the autocatalysis of precursor TPP1. Another vital residue W542 which involves in tripeptidyl peptidase activity and autocatalytic activity of TPP1 (Kuizon et al., 2010) is also obliterated when the protein is truncated after D517.

**Figure 3:**
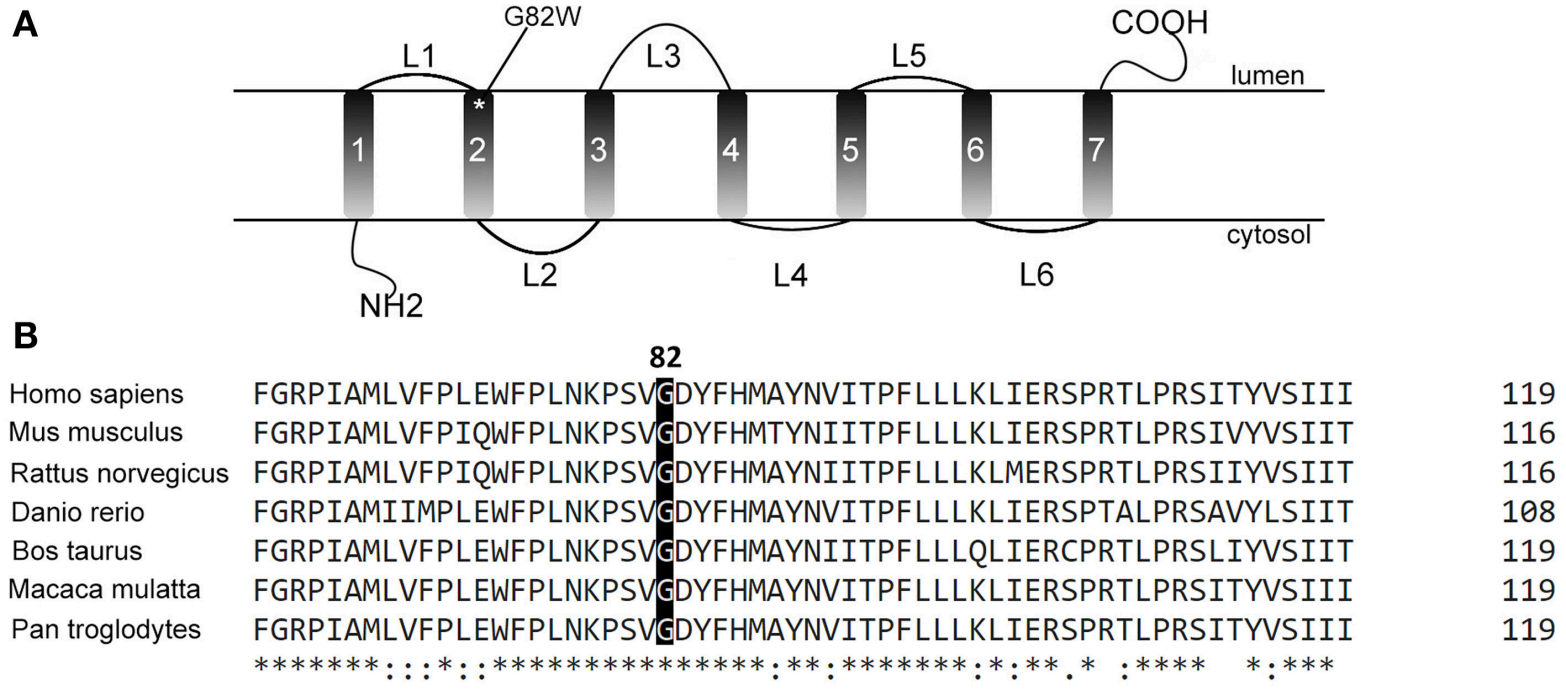
The location and conservation of residue G82. **(A)** The residue is located in the second transmembrane domain of CLN6 protein according to the previous studies. **(B)** This residue is highly conserved in various species, which indicates the importance of this residue.

Mutation c.244G>T (G82W) in CLN6 is a missense mutation which changed the nonpolar negative amino acid Glycine into a nonpolar neutral amino acid. This mutation was predicted as deleterious mutation by PolyPhen2, SIFT, PROVEAN, and MutationTaster. This result indicates that this alteration may harm the proper folding of protein and consequently affect the protein function. In addition, protein CLN6 is a transmembrane protein. The mutated residue is located on the second transmembrane domain (Figure 3A). Although the exact function and interaction of this residue is still unclear, alteration of this residue may change the anchor of CLN6 protein in lysosomal membrane. This G82 residue is highly conservative among various species (Figure 3B). It indicates that this residue is functionally important.

**Figure 4:**
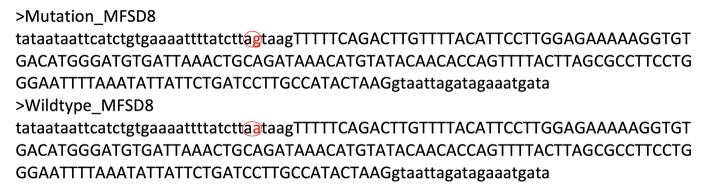
Sequences mutated MFSD8 and wildtype MFSD8. Mutation c.554-5A>G altered the “aa” sequence into an intronic splice acceptor site “ag”. This might induce splice from after the mutated “ag” site and induce four nucleotides insertion (TAAG) before the real exon thus altered all the downstream sequence.

**Figure 5:**
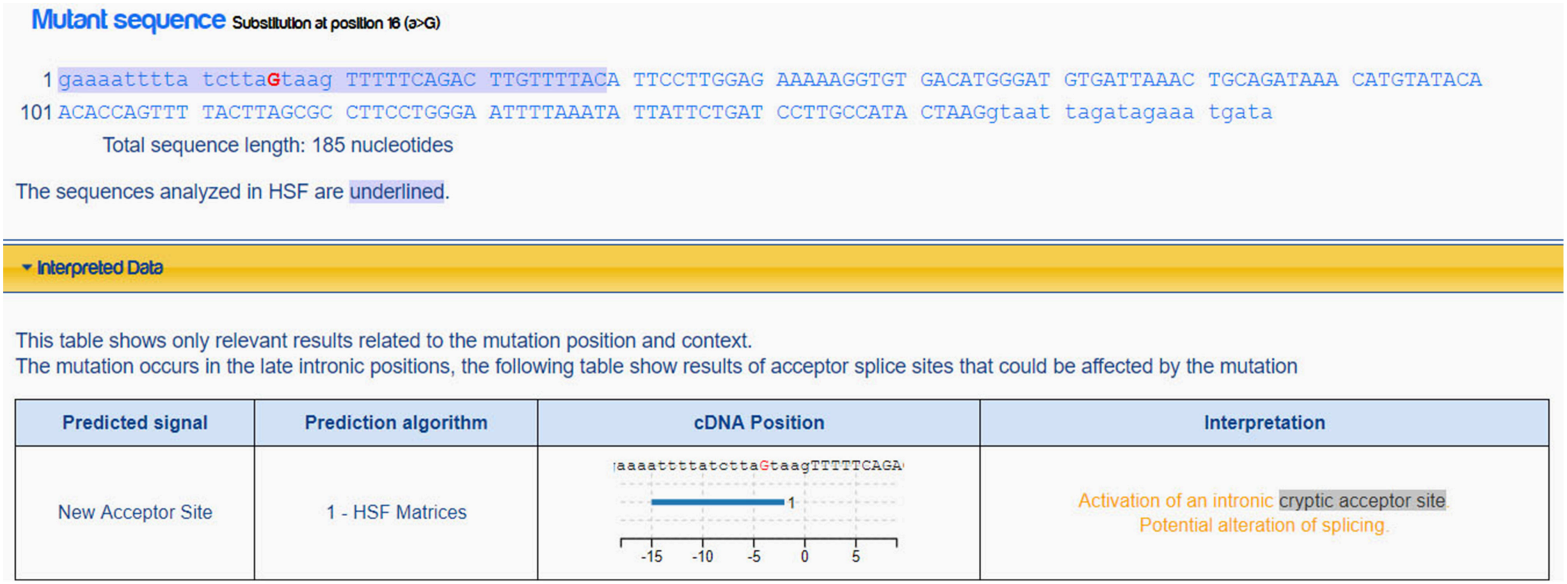
Prediction result of mutation c.554-5>G in MFSD8. This mutation would active an intronic cryptic acceptor site and potentially alter the splicing according to Human Splicing Finder Desmet et al. (2009).

Mutation c.554-5A>G, it is not a variation in canonical splice sites thus cannot be simply classified as null variant. GT/AG mRNA processing rule is valid in almost all eukaryotes including the wild type *MFSD8* sequence (Figure 4). The mutation c.554-5A>G changes the normal intronic site “aa” into another splice acceptor recognition sequence “ag” (Figure 4), which may influence the normal splicing. This hypothesis is further strengthened when predicted by Human Splicing Finder (Figure 5).

### Phenotype Study of LINCL Patients

Although CLN2, CLN5, CLN6, and CLN7 are all LINCLs, their symptoms and onset ages are slightly different in previous reports. Comparing to CLN2, clinical course of CLN5 is milder and slower, the onset age is significantly later, age of death is also significantly delayed. Onset age of visual loss in CLN5 is also significantly later than any other types of LINCLs. Reported age of death of CLN5 is around age 15 and most patients were still alive when reports were published. CLN6 was first reported as the NCL that presented similar clinical course to CLN2. The development of CLN6 was slightly slower than CLN2. Time for ambulation loss and death varied a lot. Seizures appear at the early stage in most CLN6 patients. Development of CLN7 is more severe than CLN2 as most of the patients lost ambulation within 2 years after onset, but the age of death varied from 6.5 to 18 years old. According to the previous reports, clinical information of LINCLs was summarized in Table 2 (Santavuori et al., 1982, 1991; Eva et al., 1988; Taratuto et al., 1995; Gao et al., 2002; Steinfeld et al., 2002; Sharp et al., 2003; Topcu et al., 2004; Siintola et al., 2007; Cismondi et al., 2008; Kohan et al., 2008; Aiello et al., 2009; Al-Muhaizea et al., 2009; Cannelli et al., 2009; Kousi et al., 2009; Stogmann et al., 2009; Xin et al., 2010; Perez-Poyato et al., 2012; Guerreiro et al., 2013; Patino et al., 2014; Canafoglia et al., 2015; Sato et al., 2016).

**Table 2:** Clinical information of reported cases of CLN2, CLN5, CLN6, and CLN7 before.

	**CLN2**	**CLN5**	**CLN6**	**CLN7**
Onset age	1–6 (3.1)	4.5–8	1.5–8	2–7
Initial symptoms	Motor dysfunction, speech impairment	Visual failure, psychomotor retardation, clumsiness	Mental retardation, seizures	Seizures, ataxia, motor development delay
Onset age of visual loss	4	7	4	4
Time for becoming bedridden	5	13	5~second decade	Within 2 years after onset
Time for death	End of first decade	Few died at 15	9-mid 20 s	6.5–18

Table 3 above is the clinical information of Chinese CLN2, CLN5, CLN6, and CLN7 patients mentioned in this study. The initial symptoms in this study are all involved in muscle system and motor function. The onset ages of visual loss are all slightly later than most of the reported cases. The time for becoming bedridden and death is in the range of other reported cases.

**Table 3:** Clinical information of Chinese CLN2, CLN5, CLN6, and CLN7 patients in this study.

	**CLN2**	**CLN5**	**CLN6**	**CLN7**
Onset age	8 m−3 y	4.5–5 y	3–4 y	3.5
Initial symptoms	Seizures	Unsteady giant	Unsteady giant	Unsteady giant
Onset age of visual loss	5 y	12 y	8 y	5 y
Time for becoming bedridden	6 y	12 y	15 y	5 y
Time for death	7 y	–	16 y	–

Motor function disruption then progress to intellectual function (language and cognition disorders) are predominant in the clinical course of the Chinese patients in current study. Visual system is last affected. The disease course of patients in our study is particularly consistent. Another independent report of a Chinese CLN6 patient with homozygous mutation c.892G>A (p.E298K) presented a patient with uncoordinated movements and seizures at 1.5 years, then slow response and developmental milestones were observed. Visual loss was not observed when the boy was 5 years old at the last observation (Sun et al., 2018). Other studies on CLN2 and CLN5 also presented that visual decline is never the first symptom in Chinese LINCL patients (Chang et al., 2012; Ge et al., 2018). Normal vision was found in all three Chinese CLN5 patients in the study of Ge and his colleagues (Ge et al., 2018). These studies and ours suggest that the Chinese LINCL patients may have a consistent clinical course that is slightly different from the western LINCL patients. Expanded study on Chinese patients may shed more light on the observed difference.

### Follow Up and Treatments

There is no cure in NCLs, and the treatments are limited to palliative care (Getty and Pearce, 2011). In this study, we used Topiramate to treat the CLN2 patient, Levetiracetam and sodium valproate to treat CLN5 patient, sodium valproate to treat CLN7 patient. These treatments did not generate desired outcome. We then reviewed the development of novel treatment, enzyme replacement therapy, stem cell transplantation and gene therapy.

Enzyme replacement therapy has been reported to be a more effective and safe way to treat with strong improvement observed in murine and canine models (Katz et al., 2014; Lu et al., 2015). A phase half clinical trial of the intracerebroventricular enzyme in CLN2 patients proved the safety of enzyme replacement treatment. Significant improvement of motor-language function was reported after treatment (https://www.clinicaltrials.gov/ct2/show/results/NCT01907087).

Stem cell transplantation is also considered to treat NCLs. However, hematopoietic stem cell transplantation did not perform ideally, only transient effect was observed in a few experiments (Lonnqvist et al., 2001; Yuza et al., 2005). Transplantation of neural stem cell performed better in murine model (Tamaki et al., 2009). Whereas in human, it did not change the neurological function or attenuates seizures (Selden et al., 2013).

Gene therapy was also tested. Human enzyme gene such as PPT1 or TPP1 was integrated into Adeno-Associated Virus 2 (AAV2) of AAV2/5 vector and intracranial injected into murine models or canine CLN2 models. Recombinant AAV-PPT1 successfully lowered the accumulation of autofluorescent material, increased brain mass, slowed neurodegeneration and protected behavioral functions (Griffey et al., 2004, 2006; Roberts et al., 2012; Katz et al., 2015). Safety trial for gene therapy in human is still underway (https://www.clinicaltrials.gov/ct2/show/NCT00151216).

Individual II:2 of family 4 still has not reached the onset age. Disease symptoms are not presented. These novel therapies may give positive effect on this patient as all therapies performed better at the presymptomatic stage. It certainly requires genetic test to identify causal mutation carrier in order to perform any treatment before any symptoms observed.

## Conclusion

This study described four Chinese LINCL siblings who were diagnosed by WES. The patients of these four families had similar disease courses started from motor regression or seizures to cognition regression and visual loss but carried mutations in different genes i.e. CLN2, CLN5, CLN6, and CLN7. The clinical features of LINCLs in these four Chinese siblings were not significantly different from those of Western patients. However, all Chinese LINCL patients in this study presented similar clinical course despite the affected genes. We assumed it as an ethnic specific clinical course according to our observation. Expanded sample size will be helpful to investigation of phenotype-genotype correlation. Besides, a platform for better communication, data and diagnostic experience sharing between Chinese and international clinicians is also required for further investigation (Jia and Shi, 2017).

Moreover, three mutations that detected in this study are novel mutations, and two of them occurred in intronic regions. These findings expanded the variant diversity of LINCLs.

## Ethics Statement

This study was carried out is approved by Capital Medical University Beijing Children's Hospital Ethics Committee. The protocol was approved by the Capital Medical University Beijing Children's Hospital Ethics Committee. All subjects gave written informed consent in accordance with the Declaration of Helsinki.

## Consent for Publication

The patient's parents gave written informed consent to studies and publication of clinical information, images and sequencing data.

## Author Contributions

X-TR and X-HW designed the study. X-TR, X-HW, C-HD, W-HZ, J-WL, C-HR, and FF collected the clinical information of all patients. X-TR, X-HW, and C-HD collected the follow-up and prognosis information of all patients. XS and HZ performed the Next-Generation and Sanger Sequencing. X-TR, X-HW, XS, and HZ wrote the manuscript. X-TR, XS, HZ, and C-HD revised the manuscript. All authors listed have made a substantial, direct and intellectual contribution to the work and approved it for publication.

### Conflict of Interest Statement

XS and HZ were employed by company Running Gene Inc. The remaining authors declare that the research was conducted in the absence of any commercial or financial relationships that could be construed as a potential conflict of interest.

## Supplementary Material

Figure S1 Next-Generation Sequencing results of 4 probands. **(a)** Proband 1 (Individual II:2 of family 1) is homozygous of mutation c.1551+1insTGAT. **(b,c)** Proband 2 (Individual II:2 of family 2) carries 2 mutations in CLN5 gene, c.1068_1069del **(b)** and c.1100_1103del **(c)**. **(d,e)** Proband 3 (Individual II:2 of family 3) carries 2 mutations in CLN6 gene, namely c.244G>T **(d)** and c.892G>A **(e)**. (f,g) Proband 4 (Individual II:1 of family 4) carries 2 mutations in MFSD8 gene, c.1444C>T **(f)** and c.554-5A>G **(g)**.

Figure S2 Sanger Sequencing results of 4 probands. **(A)** Individual II:2 and II:3 of family 1 are the homozygous of mutation c.1551+1insTGAT. Their parents are all carriers of this mutation and don't have the disease or any symptoms, which is corresponding to the inheritance pattern. **(B,C)** Individual II:2 and II:3 are the compound heterozygous of mutations c.1068_1069del and c.1100_1103del and meanwhile suffered from LINCL. Their father (I:1) and sister (II:1) are the carriers of mutation c.1100_1103del, their mother is the carrier of mutation c.1068_1069del. Their parent and sister are all healthy with no regression which also corresponds to the inheritance pattern. **(D,E)** Individual II:1 and II:2 of family 3 are the compound heterozygous of mutation c.244G>T and c.892G>A, and these mutations are inherited from their mother and father. Their mother is the carrier of mutation c.224G>T and their father carries mutation c.892G>A. The two carrier parents are health with no disease symptom, while 2 heterozygous children are the patients of CLN6. **(F,G)** Individual II:1 and II:2 are compound heterozygous of mutations c.1444C>T and c.554-5A>G and their parents are the carriers of these mutations, mother with c.1444C>T and father with c.554-5A>G. Two parents don't have disease, proband individual II:1 of family 4 is the patient of NCL, which is true to the heritance pattern. However, another heterozygous, individual II:2 of family 4, is asymptomatic. This is because is individual is too young and doesn't reach the onset age of CLN7.

Table S1 The precise information of patients.
