# Supplementary figures and images for: Next-Generation Sequencing Analysis Reveals Novel Pathogenic Variants in Four Chinese Siblings With Late-Infantile Neuronal Ceroid Lipofuscinosis
Source: Front Genet. 2019 Apr 25;10:370. doi: 10.3389/fgene.2019.00370 (PMC6494930; doi:10.3389/fgene.2019.00370)

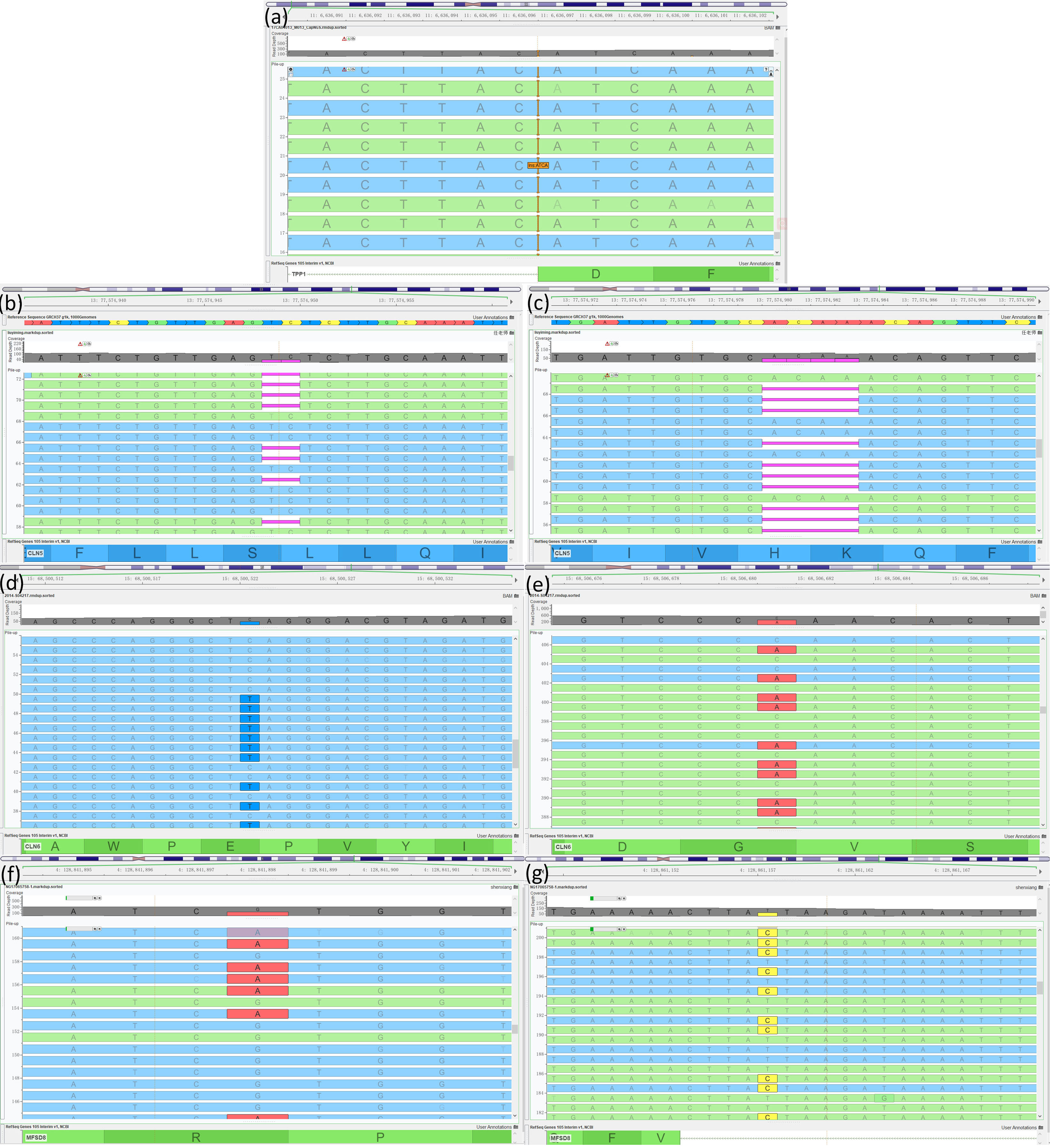

Supplement: Figure S1 — Next-Generation Sequencing results of 4 probands. (a) Proband 1 (Individual II:2 of family 1) is homozygous of mutation c.1551+1insTGAT. (b,c) Proband 2 (Individual II:2 of family 2) carries 2 mutations in CLN5 gene, c.1068_1069del (b) and c.1100_1103del (c). (d,e) Proband 3 (Individual II:2 of family 3) carries 2 mutations in CLN6 gene, namely c.244G>T (d) and c.892G>A (e). (f,g) Proband 4 (Individual II:1 of family 4) carries 2 mutations in MFSD8 gene, c.1444C>T (f) and c.554-5A>G (g). [file Image_1.JPEG]

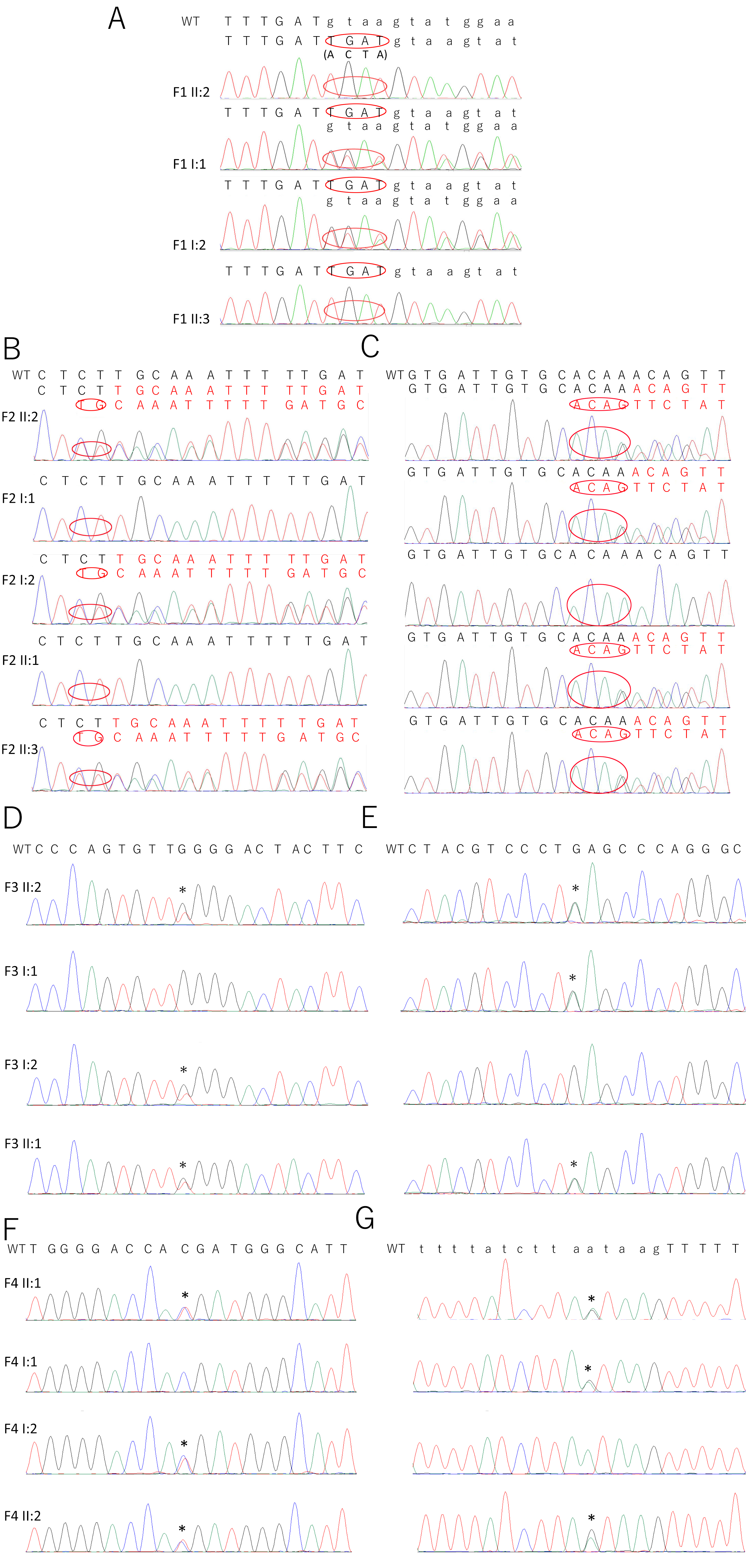

Supplement: Figure S2 — Sanger Sequencing results of 4 probands. (A) Individual II:2 and II:3 of family 1 are the homozygous of mutation c.1551+1insTGAT. Their parents are all carriers of this mutation and don't have the disease or any symptoms, which is corresponding to the inheritance pattern. (B,C) Individual II:2 and II:3 are the compound heterozygous of mutations c.1068_1069del and c.1100_1103del and meanwhile suffered from LINCL. Their father (I:1) and sister (II:1) are the carriers of mutation c.1100_1103del, their mother is the carrier of mutation c.1068_1069del. Their parent and sister are all healthy with no regression which also corresponds to the inheritance pattern. (D,E) Individual II:1 and II:2 of family 3 are the compound heterozygous of mutation c.244G>T and c.892G>A, and these mutations are inherited from their mother and father. Their mother is the carrier of mutation c.224G>T and their father carries mutation c.892G>A. The two carrier parents are health with no disease symptom, while 2 heterozygous children are the patients of CLN6. (F,G) Individual II:1 and II:2 are compound heterozygous of mutations c.1444C>T and c.554-5A>G and their parents are the carriers of these mutations, mother with c.1444C>T and father with c.554-5A>G. Two parents don't have disease, proband individual II:1 of family 4 is the patient of NCL, which is true to the heritance pattern. However, another heterozygous, individual II:2 of family 4, is asymptomatic. This is because is individual is too young and doesn't reach the onset age of CLN7. [file Image_2.JPEG]
